# Early-Onset Preeclampsia Is Associated With Gut Microbial Alterations in Antepartum and Postpartum Women

**DOI:** 10.3389/fcimb.2019.00224

**Published:** 2019-06-26

**Authors:** Li-Juan Lv, Sheng-Hui Li, Shao-Chuan Li, Zhi-Cheng Zhong, Hong-Li Duan, Cheng Tian, Hui Li, Wei He, Min-Chai Chen, Tian-Wen He, Yu-Nan Wang, Xin Zhou, Lena Yao, Ai-Hua Yin

**Affiliations:** ^1^The First Affiliated Hospital of Jinan University, Guangzhou, China; ^2^Medical Genetic Center, Guangdong Women and Children Hospital, Guangzhou, China; ^3^Promegene Institute, Shenzhen, China; ^4^Department of Obstetrics, Guangdong Women and Children Hospital, Guangzhou, China; ^5^Tianjin Key Laboratory of Cardiovascular Remodeling and Target Organ Injury, Pingjin Hospital Heart Center, Tianjin, China; ^6^Public Health Sciences Division, Fred Hutchinson Cancer Research Center, Seattle, WA, United States

**Keywords:** gut microbiota, preeclampsia, pregnancy, 16S rRNA gene sequencing, microbial dysbiosis

## Abstract

**Background:** Imbalances in gut microbiota composition are linked to hypertension, host metabolic abnormalities, systemic inflammation, and other conditions. In the present study, we examined the changes of gut microbiota in women with early-onset preeclampsia (PE) and in normotensive, uncomplicated pregnant women during late pregnancy and at 1 and 6 weeks postpartum.

**Methods:** Gut microbiota profiles of women with PE and healthy pregnant women in the third trimester and at 1 and 6 weeks postpartum were assessed by 16S rRNA gene amplicon sequencing. Plasma levels of interleukin-6 (IL-6), intestinal fatty acid-binding protein (I-FABP), zonulin, and lipopolysaccharide (LPS) were measured in the third trimesters.

**Results:** At the genus level, 8 bacterial genera were significantly enriched in the antepartum samples of PE patients compared to healthy controls, of which *Blautia, Ruminococcus2, Bilophila*, and *Fusobacterium* represented the major variances in PE microbiomes. Conversely, 5 genera, including *Faecalibacterium, Gemmiger, Akkermansia, Dialister*, and *Methanobrevibacter*, were significantly depleted in antepartum PE samples. Maternal blood pressure and liver enzyme levels were positively correlated to the PE-enriched genera such as *Anaerococcus, Ruminococcus2, Oribacterium*, and *Bilophila*, while the fetal features (e.g., Apgar score and newborn birth weight) were positively correlated with PE-depleted genera and negatively correlated with PE-enriched genera. Moreover, maternal blood IL-6 level was positively associated with gut *Bilophila* and *Oribacterium*, whereas LPS level was negatively associated with *Akkermansia*. In terms of postpartum women, both the gut microbial composition and the PE-associated microbial alterations were highly consistent with those of the antepartum women.

**Conclusion:** PE diagnosed in the third trimester of pregnancy is associated with a disrupted gut microbiota composition compared with uncomplicated pregnant women, which are associated with maternal clinical features (blood pressure level and liver dysfunction) and newborn birth weight. Moreover, these antepartum alterations in gut microbiota persisted 6 weeks postpartum.

## Introduction

Preeclampsia (PE), the second leading cause of maternal mortality worldwide (Huppertz, 2008; Ghulmiyyah and Sibai, 2012; Mol et al., 2016), is characterized by severe hypertension and multiple organ damage (Brown et al., 2018), and it can result in fetal intrauterine growth retardation, premature birth, or fetal death (Kovo et al., 2015). Untreated preeclampsia can be lethal with complications such as eclampsia, liver rupture, stroke, and kidney failure (Souza et al., 2013). In 2010, preeclampsia complicated 3–5% of pregnancies in Western countries and a higher percentage in low- and middle-income countries (Hutcheon et al., 2011; Ananth et al., 2013). Early-onset preeclampsia, which usually occurs between 20 and 34 weeks of gestation, leads to approximately 4 times more maternal death (Lisonkova et al., 2014) and 8 times more perinatal death or severe neonatal morbidity compared with mothers without preeclampsia (Lisonkova and Joseph, 2013; Khader et al., 2018). The specific etiology of preeclampsia is still unclear, and the combination of known risk factors for preeclampsia (such as women's age, body weight, previous preeclampsia, gestational hypertension, and first pregnancy) can only predict 30% of women who develop preeclampsia in clinical practice (Leslie et al., 2011; Mol et al., 2016).

Gut microbiota has profound effects on regulating host metabolism (Pedersen et al., 2016; Liu R. et al., 2017). It also plays an important role in blood pressure elevation during pregnancies (the hallmark of preeclampsia) (Gomez-Arango et al., 2016a). However, the interconnection between gut microbiota and preeclampsia is still unknown. Byproducts of gut microbial metabolism such as formate, hydrogen sulfate and toxic molecules (e.g., trimethylamine N-oxide), can directly induce elevation in blood pressure (Holmes et al., 2008; Tang et al., 2015; Tomasova et al., 2016). In contrast, the microbial flora generated short chain fatty acids can affect immune, epithelial, nervous system, and blood vessel functions to modulate blood pressure (Krautkramer et al., 2016; Schiering et al., 2017; Yan et al., 2017). Increasing evidence has revealed that gut microbiota is crucial to the development and maturation of host immune components, such as gut-associated lymphoid tissue and immunocytes (Kamada et al., 2013; Pickard et al., 2017), and also contributes to immune responses and to inhibiting inflammation (Wesemann et al., 2013). Notably, the mucosal surfaces of the gut experience low-grade inflammation with rising levels of pro-inflammatory cytokines and white blood cells as pregnancy advances, which also contributes to disturbances in intestinal flora (Koren et al., 2012). The above immune responses play a leading role in two main pathophysiological processes occurring in PE patients: (1) poor trophoblastic invasion resulted from the altered production of immunoregulatory cytokines and angiogenic factors and (2) a systemic inflammatory response (Laresgoiti-Servitje, 2013). Thus, these evidences highlight the potential and important association between gestational gut microbiota and preeclampsia.

A recent study suggested a significant shift of gut microbial composition in PE patients in late pregnancy (Liu J. et al., 2017); however, their findings remain limited due to the limitation of sample size and the lack of longitudinal investigation. Here, we conducted 16S rRNA gene sequencing to analyze the gut microbiota of 101 fecal samples of PE patients and 79 samples of healthy controls, separately in antepartum, 1 and 6 weeks postpartum. We identified the bacterial taxa associated with preeclampsia and revealed dynamic changes in the gut microbiota of patients from late pregnancy to postpartum. We also detected potential correlations between preeclampsia-associated microbes and host clinical characteristics, providing pieces for understanding the underlying mechanisms of preeclampsia.

## Methods

### Ethics Statement

This study was approved by the Ethics Committee of Guangdong Women and Children Hospital, and informed consent was obtained from all subjects in accordance with the Declaration of Helsinki (World Medical Association, 2013).

### Study Cohort and Sample Collection

All pregnant women who planned to delivery at Guangdong Women and Children Hospital from January 2017 to December 2017, were recruited for our study. After consenting and excluding those with comorbidities, multiple pregnancies, gestational diabetes and chronic hypertension, 150 singleton pregnant women with a live birth were included for analysis. In total, 78 cases newly diagnosed with preeclampsia with severe effect in their third trimesters were categorized as the PE group, while 72 normotensive and uncomplicated women were designated as the normal controls (NC group). All enrolled participants were re-invited for a follow-up visit on an average of 1 and 6 weeks postpartum. At last, a total of 101 fecal samples of the PE group (number of samples: *n* = 48, 35, and 18 at antepartum, 1 and 6 weeks postpartum, respectively) and 79 samples of the NC group (*n* = 51, 17, and 11 at the above three time points, respectively) were collected. The phenotypic characteristics of the participants are summarized in Table 1, and detailed information is given in Table S1. No smoking among participants and their family members during pregnancy was reported. All participants were of Han nationality, the ethnic majority in China. Antenatal and postnatal clinical information and pregnancy outcomes were measured and collected by well-trained staffs according to standard procedures (see below sections for details).

**Table 1:** Characteristics of the subjects with early-onset preeclampsia and normal pregnancy.

	**Antepartum**			**1 week postpartum**			**6 weeks postpartum**		
	**Normal** **control (NC)**	**Preeclampsia** **(PE)**	***P-value***	**Normal** **control (NC)**	**Preeclampsia** **(PE)**	***P-value***	**Normal** **control (NC)**	**Preeclampsia** **(PE)**	***P-value***
**No. of individuals**	51	48		17	35		11	18	
**PREGNANT WOMEN PARAMETERS**									
Age, years	29.7 ± 4	32.2 ± 5.5	0.052	29.5 ± 3.8	33 ± 5.4	0.025	29.5 ± 2.9	33.8 ± 5.4	0.036
Gestational weeks	39.8 ± 1.3	31.2 ± 4.2	< 0.001	39.4 ± 1.1	31.4 ± 4.9	< 0.001	39.7 ± 0.8	33.3 ± 6.5	0.001
Pre-preg. weight (kg)	52.7 ± 6.5	56.9 ± 10.3	0.094	49.7 ± 7.8	59 ± 12.6	0.011	53.3 ± 8.4	51.6 ± 6.6	0.617
Antenatal weight (kg)	66 ± 7.8	69.1 ± 10.3	0.261	62.6 ± 9.5	67 ± 9.4	0.202	75.5 ± 9.9	63.6 ± 8.9	0.09
Height (cm)	158 ± 4.4	158 ± 4.9	0.676	158 ± 6	158 ± 6	0.629	158 ± 6	157 ± 4	0.841
Pre-preg. BMI (kg/m^2^)	21 ± 2.5	22.8 ± 4.1	0.072	19.9 ± 3	23.3 ± 4.2	0.009	21.5 ± 3.4	20.9 ± 2.6	0.662
Fetal birth weight (kg)	3.2 ± 0.3	1.4 ± 0.7	< 0.001	3.2 ± 0.3	1.4 ± 0.9	< 0.001	3.3 ± 0.3	1.7 ± 1.2	0.026
Placenta weight (g)	544 ± 69	334 ± 129	0.001	517 ± 54	339 ± 129	0.001	523 ± 30	320 ± 170	0.032
**CLINICAL AND IMMUNE INDEXES (ANTENATAL)**									
i-FABP (pg/ml)	1090.9 ± 652.9	1450.6 ± 732.4	0.068						
zonulin (ng/ml)	19.6 ± 10.2	24.0 ± 39.7	0.553						
IL-6 (pg/ml)	6.3 ± 17.7	18.9 ± 23.6	0.031						
LPS (pg/ml)	2.9 ± 3.0	4.8 ± 4.8	0.036						
SBP (mmHg)	114 ± 12	139 ± 18	< 0.001						
DBP (mmHg)	69 ± 10	91 ± 12	< 0.001						
FBG (mmol/L)	4.4 ± 0.4	4.6 ± 0.6	0.207						
HbA1C (%)	5 ± 0.4	5.1 ± 0.5	0.597						
TG (mmol/L)	2.6 ± 1	3.8 ± 1.8	0.009						
TCH (mmol/L)	5.6 ± 1.3	6.4 ± 1.4	0.049						
HDL-C (mmol/L)	1.9 ± 0.4	1.9 ± 0.4	0.652						
LDL-C (mmol/L)	2.6 ± 0.9	3.1 ± 0.9	0.055						
HCT (%)	35.6 ± 4.2	37.1 ± 3.9	0.145						
ALT (U/L)	12 ± 14.6	18.5 ± 15.7	0.104						
AST (U/L)	16.4 ± 6.6	21.8 ± 8.6	0.014						
CRE (umol/L)	45.1 ± 7.2	62.3 ± 18.7	< 0.001						

*The data for NC and PE were presented as mean ± SD. P-values were calculated by Student's t-test. BMI, body mass index; i-FABP, intestinal fatty acid binding protein; IL-6, interleukin 6; LPS, lipopolysaccharide; SBP, systolic blood pressure; DBP, diastolic blood pressure; FBG, fasting blood glucose; HbA1C, hemoglobin A1c; TG, triglyceride; TCH, total cholesterol; HDL-C, high-density lipoprotein cholesterol; LDL-C, low-density lipoprotein cholesterol; HCT, hematocrit; ALT, alanine aminotransferase; AST, glutamic oxaloacetic transaminase; CRE, creatinine*.

The first fecal specimens (antepartum) were collected after hospitalization but before childbirth. All pregnant women delivered by cesarean section. At 1 and 6 weeks postpartum, fecal samples were collected at home by the participants, following a standardized procedure including antiseptic handling, collection in sterile tubes and immediate freezing at −20°C. The samples were then transferred to the laboratory immediately on ice and stored at −80°C until DNA extraction.

### Biochemistry and Derived Traits

Prepregnancy body weight was extracted from their pregnancy health records. Body mass index (BMI) was calculated by dividing the weight in kilograms by the square of height in meters. Office blood pressure during antepartum evaluations was measured by trained research nurses using a mercury sphygmomanometer with an appropriate cuff size, auscultating the Korotkoff sounds with the participant in the sitting position (Lei et al., 2017). Office hypertension was defined by a systolic blood pressure (SBP) ≥ 140 mmHg and/or diastolic blood pressure (DBP) ≥ 90 mmHg on three visits or by current treatment with antihypertensive medications. Preeclampsia was diagnosed according to the current guidelines (American College of Obstetricians Gynecologists Task Force on Hypertension in Pregnancy, 2013): (1) SBP/DBP ≥ 140/90 mmHg on two occasions for at least 4 h with previously normal blood pressure; (2) proteinuria ≥ 300 mg/24-h urine collection; and (3) in the absence of proteinuria, new onset of any of the following: platelet count < 100,000/μL; serum creatinine concentration > 1.1 mg/dL or a doubling in the absence of other renal disease; elevated blood concentrations of liver transaminases to twice normal concentration; pulmonary edema; and cerebral or visual symptoms. Additionally, preeclampsia with severe features was diagnosed with any of the following findings: SBP/DBP ≥ 160/110 mmHg on two occasions at least 4 h apart; platelet count < 100,000/μL; elevated blood concentrations of liver transaminases to twice normal concentration; severe persistent right upper quadrant or epigastric pain; serum creatinine concentration > 1.1 mg/dL or a doubling in the absence of other renal disease; pulmonary edema; and new-onset cerebral or visual disturbances.

Fasting blood and 24-h urine samples in parallel to the BP monitoring were performed. Biochemical measurements included fasting plasma glucose level, plasma levels of total cholesterol, high density lipoprotein cholesterol (HDL), low density lipoprotein cholesterol (LDL), triglycerides, creatinine, 24-h urinary protein excretion, interleukin-6 (IL-6), intestinal fatty acid-binding protein (I-FABP), zonulin, and lipopolysaccharide (LPS). In the second visit, which was performed 1 week after childbirth, the subjects' body weight, blood pressure and diet questionnaire were also recorded. Plasma IL-6 (Thermo/eBiosecience) concentrations were quantified using a double antibody sandwich enzyme-linked immunosorbent assay (ELISA). Plasma I-FABP (R&D Systems) concentrations were quantified by specific ELISA. Zonulin in plasma was estimated by competitive ELISA (Alpha Diagnoestic International). Plasma LPS (antibodies online) was estimated by sandwich ELISA.

### DNA Extraction and 16S rRNA Gene Sequencing

The microbial genomic DNA was extracted according to the MOBIO PowerSoil® DNA Isolation Kit 12888-100 protocol, and DNA was stored at −80°C in Tris-EDTA buffer solution before use. To enable amplification of the V4 region of the 16S rRNA gene and add barcode sequences, unique fusion primers were designed based on the universal primers set, 515F (5′-GTGYCAGCMGCCGCGGTAA-3′) and 806R (5′-GGACTACNVGGGTWTCTAAT-3′), along with barcode sequences. PCR mixtures contained 1 μL of each forward and reverse primer (10 μM), 1 μL of template DNA, 4 μL of dNTPs (2.5 mM), 5 μL of 10 × EasyPfu Buffer, 1 μL of Easy Pfu DNA Polymerase (2.5 U/μL), and 1 μL of double distilled water in a 50 μL reaction volume. Thermal cycling consisted of an initial denaturation step at 95°C for 5 min, followed by 30 cycles of denaturation at 94°C for 30 s, annealing at 60°C for 30 s, and extension at 72°C for 40 s, with a final extension step at 72°C for 4 min. Amplicons from each sample were run on an agarose gel. The expected band size for 515F-806R is ~300–350 bp. Amplicons were quantified with the Quant-iT PicoGreen dsDNA Assay Kit (ThermoFisher/ Invitrogen cat. no. P11496; following the manufacturer's instructions).

The amplicon library was combined in equal amount and subsequently quantified (KAPA Library Quantification Kit KK4824) according to the manufacturer's instructions, and high-throughput sequencing on the Illumina MiniSeq platform at Promegene Co. Ltd. (Shenzhen, China) was performed to generate 150 bp paired-end reads (exclude the primer sequences) for each sample.

### Microbiome Analyses

Raw sequencing reads were eliminated from analysis if they produce >8 homopolymers, >2 mismatches in the primers, or >1 mismatches in the barcode. High-quality paired-end sequencing reads were analyzed based on the quantitative insights into microbial ecology (QIIME2, https://qiime2.org/) platform (Kuczynski et al., 2012), and the standard tools/plugins provided by QIIME2. First, raw 16S sequences were performed for quality control and to feature table construction using the DADA2 algorithm (Callahan et al., 2016). Possible phiX reads and chimeric sequences were removed, and the remaining reads were truncated from 0 to 140 bases (for both forward and reverse reads) to avoid the sequencing errors at the end of the reads. Paired-end reads were overlapped at the maximum mismatch parameter of 6 bases, which means a minimum similarity threshold of 90% on the overlap zone of the forward and reverse reads. The representative sequences (named “feature” in QIIME2 nomenclature) were then generated by removing the redundant and low occurrence (*n* < 5 in pool samples) sequences. We used the term “operational taxonomic unit (OTU)” instead of “feature” in the whole article for convenience. Then, taxonomic assignment of the OTUs were determined based on a pretrained Naive Bayes classifier (trained on the Greengenes 13_8 99% OTUs DeSantis et al., 2006) via the q2-feature-classifier plugin, and the taxonomic composition at the phylum, class, order, family, genus, and species levels were generated based on OTU annotation. To avoid sampling depth bias, 20,000 reads were randomly selected from each sample when calculating the OTU and taxa relative abundances.

Phylogenetic analyses were realized via the q2-phylogeny plugin, which performed multiple sequence alignment on the OTU sequences and generated phylogenetic trees of the OTUs from the alignment result. Four estimators of the alpha diversity, including Shannon's diversity index, observed OTUs, Faith's phylogenetic diversity (a qualitative measure of community richness that incorporates the phylogenetic relationships between the OTUs) and Pielou's evenness, and four estimator of the beta diversity, including Jaccard distance, Bray-Curtis distance, unweighted UniFrac distance, and weighted UniFrac distance, were used in this study and calculated based on the QIIME2 q2-diversity plugin.

Enterotype of the fecal samples were determined based on their genus level composition using a reference-based alignment algorithm (http://enterotypes.org/) (Costea et al., 2018).

Functional composition of the samples was generated using the PICRUSt2 algorithm (Langille et al., 2013). For each sample, the composition of the Kyoto Encyclopedia of Genes and Genomes (KEGG) (Kanehisa et al., 2017) orthologs (KOs) was predicted based on the functional information of the reference OTUs. KEGG modules and pathways composition were generated according to the assignment of KOs at https://www.kegg.jp/.

### Statistical Analyses

Statistical analyses were implemented at the R v3.3.2 platform (https://www.r-project.org/). Permutational multivariate analysis of variance (effect size analysis) was performed with the *adonis* function of the R *vegan* package (https://cran.r-project.org/web/packages/vegan/index.html), and the *adonis P*-value was generated based on 1,000 permutations. Distance-based redundancy analysis (dbRDA) was performed on the OTUs and taxonomic composition profiles with the *vegan* package, based on the Bray-Curtis distance, and visualized via the R *ade4* package. The PE-associated OTUs and taxa were identified based on the Wilcoxon rank-sum test. Random forest models were analyzed with the R *randomForest* package (1,000 trees). The performance of the predictive model was evaluated with the leave-one-out cross validation. Receiver operator characteristic (ROC) analysis was performed using the R *pROC* package. The ROC curve was created by plotting the true positive rate (sensitivity) against the false positive rate (1- sensitivity), and the area under the curve (AUC) was calculated to assess the ensemble. Procrustes analysis was performed with the R *vegan* package, and the Procrustes *P*-value was generated based on 1,000 permutations. *P*-value < 0.05 was considered statistically significant. The *q* was used to evaluate the false discovery rate for correction of multiple comparisons, and was calculated based on the R *fdrtool* package.

## Results

### Study Cohort and Sequencing Summary

To evaluate the associations between PE and the composition of women's gut microbiome in the perinatal/postnatal period, we analyzed the fecal samples of PE patients and healthy controls at antepartum (*n* = 48 vs. 51), 1 week postpartum (*n* = 35 vs. 17), and 6 weeks postpartum (*n* = 18 vs. 11). Patients and controls were matched according their body weight parameters including prepregnancy weight, antenatal weight, height and BMI at each time point (Table 1); on average, the PE patients were 3 years older than the controls (*P* < 0.05 at 1 and 6 weeks postpartum). PE patients and healthy controls were differed in their PE-associated clinical status at antepartum (Table 1) and after delivery (Table S1). Based on their self-reports and questionnaires, all participants were similar in their dietary habit and lifestyle (data not shown). To avoid the effect of delivery mode on gut microbiota, only pregnant women with cesarean section were included in this study.

**Figure 1:**
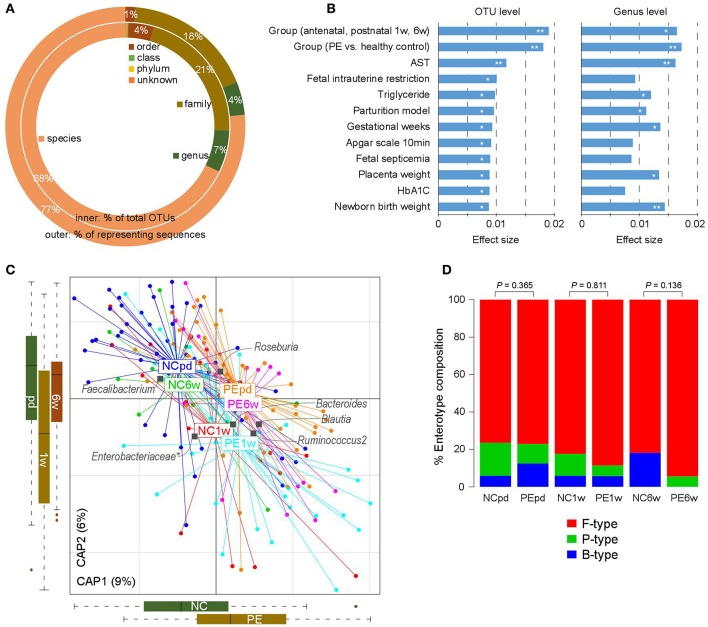
Overview of the gut microbial community in PE patients and healthy controls. **(A)** Summary of the taxonomic assignment of the OTUs. Inner circle, the percentage of OTUs that assigned into a taxon at the species, genus, family, order, class, and phylum levels. Outer circle, the percentage of representing sequences of the OTUs at all taxonomic levels. **(B)** Host factors that significantly affected the gut microbiota. The 12 factors associated with the variation of the gut microbiota at the OTU level are shown. Bar plots indicate the explained variation (effect size) of each factor at the OTU level (left panel) and genus level (right panel). **, permutated *P* < 0.01; *, permutated *P* < 0.05. **(C)** dbRDA plot based on the Bray-Curtis distances between microbial genera, revealing PE microbial dysbiosis at the antepartum, 1 and 6 weeks postpartum periods. Patient and control samples were mainly separated in the primary constrained axis (PE separation), and samples at different time points were mainly separated in the second constrained axis (time point separation). Lines connect samples (colored points) in the same group, and circles cover samples near the center of gravity for each group. Genera (yellow squares) as the main contributors are plotted by their loadings in these two components. **(D)** Constitution of enterotypes in all groups. *P*-values between PE patients and healthy controls at three time points were calculated based on Fisher's exact test.

The gut microbiotas of 180 fecal samples of pregnant women were characterized by sequencing the V4 variable region of the bacterial 16S rRNA gene, generating a total of 8,136,758 high quality sequences (45,204 ± 11,017 per sample; Table S1). A total of 2,293 OTUs were identified and taxonomically annotated based on an open source, universal microbiome bioinformatics platform, QIIME2 (Kuczynski et al., 2012). Of which, 74% could be annotated into specific genus (representing 81% of total sequences; Figure 1A), and 68% could be annotated into specific species (representing 77% of total sequences).

### Diversity and Overall Structure of Gut Microbiota

The intrasample richness and intersample relationship of the gut microbiota of all participates were assessed by alpha and beta diversity indexes, respectively. No significant differences in alpha diversity were detected during the antepartum and postpartum periods (*P* > 0.05 for all four indexes of alpha diversity; Figure S1). Similarly, the beta diversity between different groups did not differ significantly (*P* > 0.05 for all four indexes of beta diversity).

We then tested if PE was associated with the holistic community structure over the entire cohort. PE accounted for 1.8% (*adonis P* < 0.001) and 1.7% (*adonis P* = 0.002) of the gut microbiota variance at the OTU and genus levels, respectively. This effect size was relatively larger than other collected confounding factors, including the intrinsic parameters of pregnant women, fetal features, and antenatal clinical immune indexes (Figure 1B; Table S2), indicating that PE stratification was one of the main reasons in our cohort. Likewise, the grouping of antenatal, 1 and 6 weeks postpartum also accounted for an approximate proportion of microbial variances (1.9% at the OTU level and 1.6% at the genus level) with PE.

Bray-Curtis distance based redundancy analysis (dbRDA, analyzed at the genus level) captured visible separation of both PE stratification and sample time grouping on the overall gut microbiota (Figure 1C). PE significantly acted on the primary constrained axis of the dbRDA plot, while the genera *Faecalibacterium, Blautia*, and *Ruminococcus2* represented the major contributors in the axis. Likewise, sample time grouping affected the second constrained axis, while *Roseburia, Enterobacteriaceae*, and *Ruminococcus2* were the major contributors. Noticeably, despite the samples at 1 week postpartum were separated with others, the antenatal and 6 weeks postpartum samples were closely related in the second constrained axis, suggesting a remarkable shift of gut microbiota at 1 week postpartum but the majority recovered by 6 weeks postpartum.

The primary structure of the human gut microbiome is described by enterotypes (Costea et al., 2018). In our samples, the enterotype stratification was clearly driven by the abundance of several dominate genera such as *Bacteroides* (B-type), *Prevotella* (P-type), and *Ruminococcus* (F-type). PE patients exhibited consistent enterotype patterns with healthy controls from late pregnancy to postpartum (Figure 1D), despite the PE samples seeming to have a slight increase of F-type and decrease of B-type.

### Microbial Taxa Signatures

**Figure 2:**
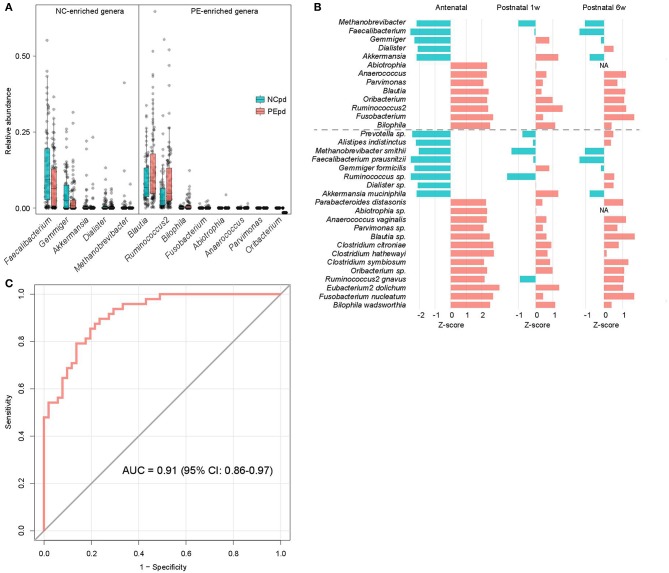
Difference of gut microbiota between PE patients and healthy controls. **(A)** Boxplot shows the significantly different genera between patients and controls. Genera with *P* < 0.05 (Wilcoxon rank-sum test) of samples at antepartum are shown. The boxes represent the interquartile range (IQR) between the first and third quartiles and the line inside represents the median. The whiskers denote the lowest and highest values within 1.5 times IQR from the first and third quartiles, respectively. **(B)** The PE-associated genera and species of samples at antepartum, and their tendencies in samples at 1 and 6 weeks postpartum. The bar lengths indicate the Z-score of a genus or species at different time points, and the colors represent enrichment in patients (red, Z-score > 0) or controls (blue, Z-score < 0). **(C)** ROC analysis for classification of PE status by the relative abundances of PE-associated genera, assessed by AUC.

We compared the gut microbial composition of PE patients and healthy controls at each time point to investigate microbial signatures of PE. At the phylum level, PE patients had a similar composition in the dominant phyla compare to the healthy controls, except several low abundant phyla (Fusobacteria, Tenericutes, and Verrucomicrobia) were significantly depleted in PE patients at the antenatal time point (Table S3). At the genus level, 8 genera were significant enriched in antenatal PE samples (Figure 2A), of which *Blautia* and *Ruminococcus2*, followed by *Bilophila* and *Fusobacterium* represented the major variances in PE microbiomes. The main species-level members of these genera, including an unclassified *Blautia* spp. (consisted 84.9% of *Blautia*), *R. gnavus* (consisted 54.7% of *Ruminococcus2*), *B. wadsworthia* (consisted 100% of *Bilophila*), and *F. nucleatum* (consisted 100% of *Fusobacterium*), were also significantly increased in PE patients (Table S3). Inversely, 5 genera, including *Faecalibacterium, Gemmiger, Akkermansia, Dialister*, and *Methanobrevibacter*, were significantly depleted in antenatal PE samples, which mostly consisted of the species *F. prausnitzii* (100%), *G. formicilis* (100%), *A. muciniphila* (100%), an unclassified *Dialister* spp. (100%), and *M. smithii* (100%), respectively. Moreover, several genera and species were significantly altered between the PE and control microbiota at 1 and 6 weeks postpartum (Table S3).

The majority of taxonomic alterations in antenatal PE microbiota pertains to 1 or 6 weeks postpartum when 79.4% and 84.4% of antenatal-altered clades (at the genus and species levels) were within concordant tendency in samples of 1 and 6 weeks postpartum samples (Figure 2B), respectively. This finding indicated that the antenatal-altered taxa might be reliable microbial markers for PE across the antenatal and postnatal stages.

Using the random forest model, we evaluated the performance of gut microbial composition to predict PE status based on the relative abundance of 13 antenatal-altered genera. The model achieved an AUC of 0.91 (95% confidence interval, 0.86–0.97) for the discrimination of PE patients and healthy controls in antenatal samples (Figure 2C).

### PE-Associated Microbes Correlate to Clinical and Immune Parameters

**Figure 3:**
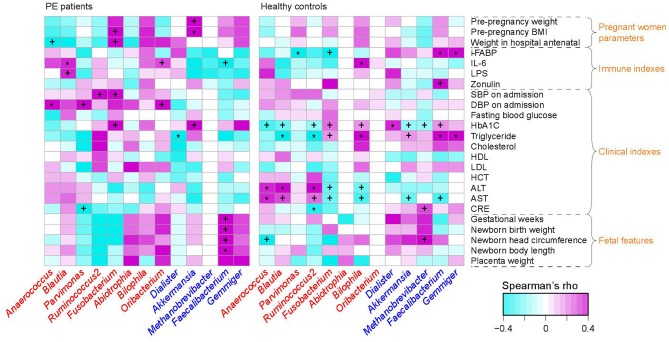
Correlation between PE-associated genera and the host parameters. The heatmap panel shows the Spearman correlation coefficient between the genera (text color: red, enriched in PE patients; blue, enriched in healthy controls) and host parameters. Significance levels in correlation tests are denoted: +,*q* < 0.10; *,*q* < 0.05.

To investigate the interassociations between gut microbial composition and host clinical status, we identified statistical correlations between 13 PE-associated genera and the pregnant women's parameters, including blood indexes, fetal features, and antenatal clinical immune indexes. Significant associations were observed in the separate patient and control groups (Figure 3). For example, in PE patients, the women's DBP and SBP levels were positively correlated with PE-enriched genera, such as *Anaerococcus, Ruminococcus2, Fusobacterium*, and *Oribacterium*, while the fetal features (e.g., birth weight) were positively correlated with PE-depleted genera. One of the pregnant women's immune parameters, IL-6 was positively associated with *Blautia* (ρ = 0.36, *q* = 0.04; in PE patients) and *Bilophila* (Spearman's ρ = 0.37, *q* = 0.01; in healthy controls), and negatively associated with *Faecalibacterium* (ρ = −0.27, *q* = 0.04; in PE patients).

### KEGG Functional Signatures

**Figure 4:**
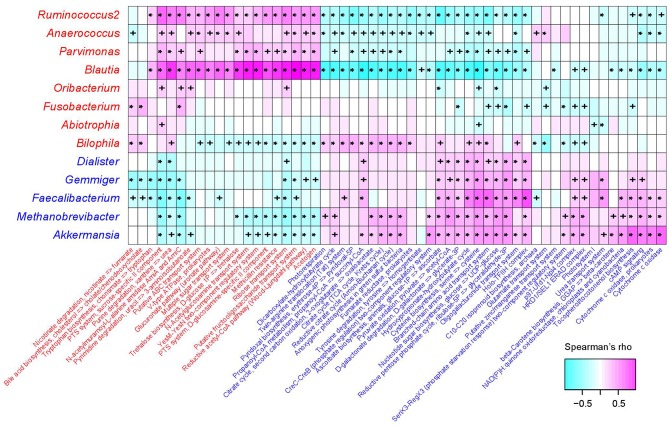
Correlation between PE-associated genera and functional modules. The heatmap panel shows the Spearman correlation coefficient between the genera and KEGG functional modules. Text color for genera and modules: red, enriched in PE patients; blue, enriched in healthy controls. Significance levels in correlation tests are denoted: +,*P* < 0.05; *,*P* < 0.01.

The functional capacity of the gut microbiota of PE patients and healthy controls were predicted by PICRUSt analysis (Langille et al., 2013) based on their 16S OTU profiles, which led us to quantify the relative abundance of 494 KEGG modules. In all, 66 modules (13.4%) significantly differed in their abundance between PE patients and healthy controls (*q* < 0.05; Table S4), including 46 PE-reduced and 20 PE-enriched modules. The majority of the PE-reduced modules involved to central and other carbohydrate metabolism (*n* = 9, including 2 terpenoid biosynthesis modules), carbon fixation (*n* = 4), amino acid, cofactor, and vitamin metabolism (*n* = 5), ATP synthesis and photosynthesis (*n* = 7), two-component regulatory system (*n* = 8), and the transport systems of several small molecules (*n* = 7), while the PE-enriched modules involved various functional sets including 4 saccharide transport systems. Thirty-six (78.3%) PE-reduced modules, as well as all the PE-enriched modules, were significantly correlated with one or more PE-associated genera (Figure 4). In PE-enriched genera, *Blautia* and *Ruminococcus2* were associated with the majority of PE-enriched functional modules, suggesting their central role in the PE microbiome. In addition, *Fusobacterium* and *Bilophila* had complementary roles, as they were uniquely positively correlated with the adverse modules involved in bile acid biosynthesis and nicotinate degradation. In PE-reduced genera, *Faecalibacterium, Methanobrevibacter*, and *Akkermansia* played central roles.

### Longitudinal Variation of Gut Microbial Diversity and Composition

**Figure 5:**
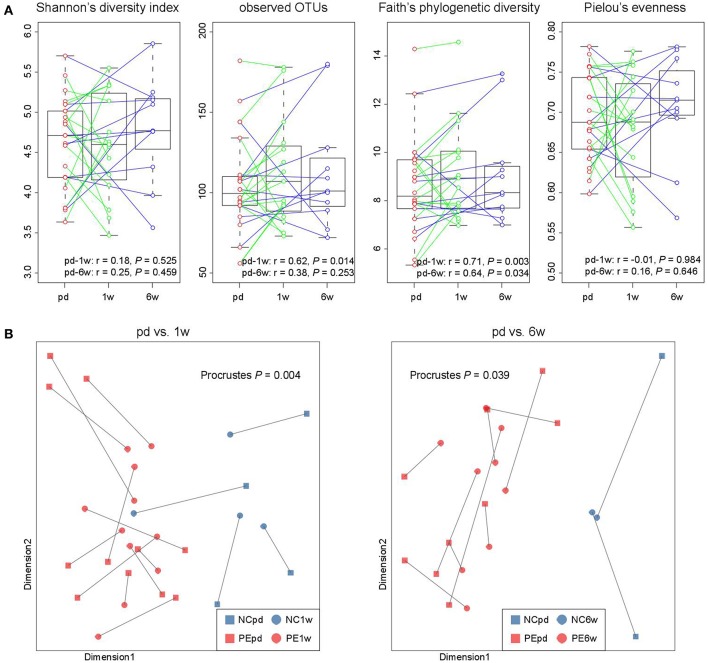
Longitudinal comparison of gut microbial diversity and composition between antepartum, 1 and 6 weeks postpartum. **(A)** Change in alpha diversity from the antepartum to 1 and 6 weeks postpartum. The boxes represent the interquartile range (IQR) between the first and third quartiles and the line inside represents the median. The whiskers denote the lowest and highest values within 1.5 times IQR from the first and third quartiles, respectively. Pearson correlation coefficient of samples between two time points was calculated, and *P*-value was tested using the correlation test. **(B)** Procrustes analysis reveals the covariation between samples at the antepartum, 1 and 6 weeks postpartum periods.

In our cohort, the fecal samples of 15 women were collected at antepartum and 1 week postpartum, and 11 women provided samples at antepartum and 6 weeks postpartum, which allowed us to investigate the longitudinal changes. From antepartum to 1 week postpartum, the observed OTUs and phylogenetic diversity (two indexes of microbial richness) in women's gut microbiota were consistent (Figure 5A), but their microbial evenness was disturbed. From antepartum to 6 weeks postpartum, all microbial alpha diversity indexes were correlated between the two time points. With regard to the aspect of microbial composition, we found that samples at both 1 and 6 weeks postpartum were highly consistent with samples at antepartum (Figure 5B; *P* < 0.05 for two comparisons).

## Discussion

In this study, the changes of gut microbiota of pregnant women with PE before and after delivery were analyzed and compared with those of normotensive, uncomplicated pregnant women in the antepartum, 1 and 6 weeks postpartum periods. Our study demonstrated that the composition of gut microbiota in PE patients remarkably differed from that in normal pregnant women throughout the evaluation periods.

Currently, the exact cause of PE remains elusive. Previous studies have suggested that metabolic abnormalities, systemic inflammatory reactions, oxidative stress, and vascular endothelial damage are involved in the pathogenesis of PE (Catalano et al., 2012; Paauw et al., 2016). Our study suggested that gut microbiota may also be one of the participants. Similar studies on gestational hypertension and gestational diabetes mellitus (GDM) supported the hypothesis that gestational diseases are associated with changes in gut microbiota (Kuang et al., 2017; Crusell et al., 2018). Gomez-Arango et al. (2016a) found that the abundance of *Odoribacter*, a bacteria producing butyric acid, was negatively correlated with the SBP of pregnant women at 16 weeks of pregnancy. Crusell et al. (2018) found that GDM was associated with a change in the gut microbiota composition in both the third trimester of pregnancy and postpartum. They especially reported an enrichment in the abundance of *Blautia* and *Ruminococcus2* in diabetic patients, which was also observed in the PE patients in our cohort. Studies on reducing the risk of PE through probiotic supplementation (Brantsaeter et al., 2011) confirmed that PE is associated with gut microbes. For years, the strategies for managing PE have been limited to symptomatic therapy or the termination of pregnancy (Xia and Kellems, 2013). This suggests that regulating intestinal microbiota through probiotics may play a role in the prevention of PE. Thus, exploring the changes and functions of intestinal microbiota in PE may provide new ideas for the prevention and treatment of PE.

We found that PE stratification accounted for nearly 2% of gut microbial variation (Figure 1B), which was relatively larger than other host parameters. As revealed by previous population-based reports (Falony et al., 2016; Zhernakova et al., 2016), a large proportion (80–90%) of variation of human gut microbiome was explained by unexplained or intrinsic factors (e.g., enterotypes), whereas the host parameters including various kinds of diseases only explained a limited proportion of variations (usually <1%). In addition, we identified 13 PE-associated genera (Figure 2A), which achieved a higher discriminatory power (AUC = 0.91) for discriminating PE and control samples at the antenatal stage (Figure 2C) compared to that of other diseases such as diabetes (Qin et al., 2012) and hypertension (Yan et al., 2017). Thus, these findings highlighted the remarkable dysbiosis of gut microbiota in PE patients.

**Figure 6:**
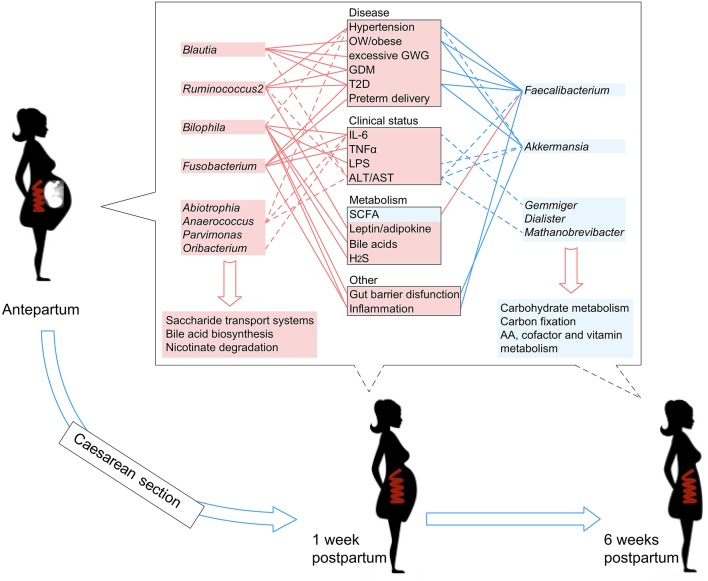
A schematic diagram showing the PE-associated bacteria and functions that had predicted effects on the host disease, clinical status, and metabolism. The PE-enriched species and functions are shown in the left red columns, and the PE-depleted species and functions are shown in the right blue columns. Center boxes show the host properties. Lines connect the PE-associated species and host properties with potential associations, while the dotted lines indicate the correlations which were identified in this study.

Literature searches of the 13 PE-associated genera showed that these bacteria were also associated with other host diseases including obesity, higher glucose metabolic disorders, pro-inflammatory effects, intestinal barrier dysfunction, and bile acid dysmetabolism (see discussion below). In addition, these microbes were able to be correlated with host immune parameters and function markers, such as IL-6 and LPS (Signat et al., 2011; Hunter and Jones, 2015), and such correlations were also found in the current study (Figure 3A). Overall, these findings suggested that PE patients harbor an inflammation-associated microbiota. The schematic of PE-microbiota is summarized in Figure 6, and the potential relevance of mechanisms is described below.

In PE-enriched genera, *Blautia* and *Ruminococcus2* were associated with the majority of PE-enriched functional modules, suggesting their central role in the PE microbiome. Members of the genus *Blautia* are generally gram-positive bacteria that produce acetate, ethanol, hydrogen, lactate, or succinate as the end products of glucose fermentation (Liu et al., 2008). *Blautia* has been associated with obesity and type 2 diabetes (Cani et al., 2012; Kasai et al., 2015), and is enriched in prepregnancy overweight/obese and excessive gestational weight gain women (Stanislawski et al., 2017) and glucose-intolerant individuals (Egshatyan et al., 2016). These findings are in line with the results of Crusell et al. (2018) who showed increased abundances associated with GDM, suggesting that enriched *Blautia* abundance goes together with a nonfavorable metabolic profile. Similarly, *Ruminococcus2* was also enriched in women with GDM (Zacarías et al., 2018) as well as type 2 diabetic patients (Zhang et al., 2013), and *R. gnavus* has been reported to be enriched in people with dysmetabolism and low microbial gene count (Le Chatelier et al., 2013). In addition, Ruminococcaceae are strongly correlated with the leptin level (an adipocyte-derived hormone that plays a direct role in the pathogenesis of PE) in the human body (Miehle et al., 2012; Taylor et al., 2015; Gomez-Arango et al., 2016b). Furthermore, we revealed that *Ruminococcus2* abundance in the intestinal tract of PE patients was positively correlated with SBP/DBP on admission and ALT/AST levels in pregnant women (Figure 3A), suggesting that the *Ruminococcus2* may also be directly related to the incidence of PE via its effect on the host's blood pressure or liver function.

*Bilophila* (*B. wadsworthia*) and *Fusobacterium* (*F. nucleatum*) also represented the major variance in PE microbiomes. *B. wadsworthia* can promote higher inflammation via producing hydrogen sulfide (da Silva et al., 2008), intestinal barrier dysfunction and bile acid dysmetabolism (Devkota et al., 2012; Natividad et al., 2018). Moreover, higher amounts of *B. wadsworthia* can release LPS and IL-6 (Hunter and Jones, 2015), which is in agreement with the observation in this study showing a positive correlation between the prenatal plasma IL-6 level and the abundance of intestinal *Bilophila*. *F. nucleatum* is one of the most prevalent gut and oral species that is associated with a wide spectrum of human diseases such as adverse pregnancy outcomes (e.g., preterm birth and neonatal sepsis) (Barak et al., 2007; Han et al., 2010; Bohrer et al., 2012; Wang et al., 2013) and gastrointestinal tract disorders (e.g., colorectal cancer and inflammatory bowel disease) (Han, 2015). The virulence mechanisms include its colonization, systemic dissemination, and induction of host inflammatory and tumorigenic responses (Han, 2015). Additionally, *F. nucleatum* is also a potent stimulator of inflammatory cytokines, such as IL-6, IL-8, and TNFα (Han et al., 2000; Park et al., 2014).

Except for the PE-enriched bacteria, several species including *Faecalibacterium, Methanobrevibacter*, and *Akkermansia* were depleted in PE patients. *Faecalibacterium*, one of the most abundant and important commensal bacteria in the human gut microbiota, produces short-chain fatty acids (SCFAs, especially butyrate) (Machiels et al., 2014), and protects the intestines, and is involved the reduction of obesity, diabetes and inflammation (Sokol et al., 2008; Miquel et al., 2013). Lower production capacity of SCFAs may therefore contribute to higher blood pressure, and thus increase the risk of PE in pregnant women (Pevsner-Fischer et al., 2017; Yan et al., 2017). In addition, *Akkermansia muciniphila* is associated with a low risk of diabetes (Shin et al., 2014), obesity (Everard et al., 2013), and high inflammation (Ganesh et al., 2013), based on its unique mucin-degrading capacity that functions to strengthen the integrity of the host's gut barrier (Guo et al., 2017).

Despite the pregnant women's gut microbiota being largely separated by the division of three time stages, the microbial alterations of the PE microbiome at the antenatal stage could also reflect on the samples at 1 and 6 weeks postpartum (Figure 2B). Particularly, but not significantly, the gut microbiomes of samples at 6 weeks postpartum were closer to the antenatal samples than that of the 1 week postpartum samples, either in the whole microbial composition (Figure 1C) or in the PE-associated taxa (Figure 2C). A similar phenomenon was also revealed in previous studies showing that the women's gut microbial structure dramatically changed during delivery and sustained recovery after a long period of time (DiGiulio et al., 2015; Wang et al., 2018). As a supplement, our intraindividual samples of antepartum-1 week-postpartum pairs and antepartum-6 weeks-postpartum pairs also showed highly microbial diversity and compositional consistency (Figure 5).

One of the limitations of the current study was sample size in postpartum for both week 1 and 6. The lack of close matching in individual confounding factors, such as genetic background, host geography, diet and lifestyle could also limit interpretations of this study results. Another limitation was that samples of women during 1st or 2nd trimester were not included in the study. Thus, our study could not suggest any causal relationship between the altered gut microbiota and PE pathogenicity. Future studies with a larger cohort from early pregnancy to postpartum will be needed to further understand the relationship between gut microbiome and PE. Testing specific microbiota in animal models is also beneficial to elucidate the mechanism of interaction between gut microbiome and preeclampsia during pregnancy.

## Conclusion

To our knowledge, this is the first study investigating the gut microbiota composition in PE patients at both the time points of antepartum and postpartum. Our findings add more important information of correlation between gut microbiota and PE and extend the previous knowledge. The taxonomic signatures, microbe-clinical associations, and function signatures identified in this study suggested possible pathways for PE pathogenicity and provided potential markers for PE prediction and intervention.

## Data Availability

The raw sequencing dataset acquired in this study has been deposited to the European Bioinformatics Institute (EBI) database under the accession code PRJEB33074 (https://www.ebi.ac.uk/ena/data/view/PRJEB33074). The sample metadata, OTU and taxonomic composition data, and the statistical scripts are available from the corresponding author on reasonable request.

## Author Contributions

A-HY substantially contributed to the conception of the work, experimental, and manuscript guidance. L-JL mainly carried out the cohort research performance, data analysis, and drafted the manuscript. S-HL was responsible for statistics, analysis, and functional annotation of bioinformatics data. S-CL, CT, and Z-CZ participated in the implementation of the experiment. H-LD, HL, WH, M-CC, T-WH, and Y-NW were responsible for samples storage and fellow-up of the cohort. S-HL, XZ, and LY critically revised the manuscript for important intellectual content. All the participants provided approval for publication of the content, agreed to be accountable for all aspects of the work in ensuring that questions related to the accuracy or integrity of any part of the work are appropriately investigated and resolved.

### Conflict of Interest Statement

The authors declare that the research was conducted in the absence of any commercial or financial relationships that could be construed as a potential conflict of interest.

## Supplementary Material

Table S1 Basic information and sequencing summary of 180 analyzed samples.

Table S2 PERMANOVA (adonis) analysis of host parameters on their gut microbiotas. Analysis were performed on the OTU and genus profiles to test whether the individuals' parameters have significant impact on the gut microbiota meta.

Table S3 Detailed information of the PE-associated taxa at the phylum, genus and species levels. Genera with *P* < 0.05 (Wilcoxon rank-sum test) at antepartum, 1 or 6weeks postpartum are shown.

Table S4 Detailed information of the PE-associated functional modules. Note: green, significantly enriched in healthy controls; red, significantly enriched in PE patients.

Figure S1 Comparison of alpha and beta diversity between PE patients and healthy controls. **(A)** Difference of four alpha diversity indexes between patients and controls. The boxes represent the interquartile range (IQR) between the first and third quartiles and the line inside represents the median. The whiskers denote the lowest and highest values within 1.5 times IQR from the first and third quartiles, respectively. **(B)** PCoA plot based on four beta diversity indexes. The first and second principal coordinates are shown. Lines connect samples (colored points) in the same group, and circles cover samples near the center of gravity for each group.
