# Supplementary figures and images for: Early-Onset Preeclampsia Is Associated With Gut Microbial Alterations in Antepartum and Postpartum Women
Source: Front Cell Infect Microbiol. 2019 Jun 26;9:224. doi: 10.3389/fcimb.2019.00224 (PMC6608563; doi:10.3389/fcimb.2019.00224)

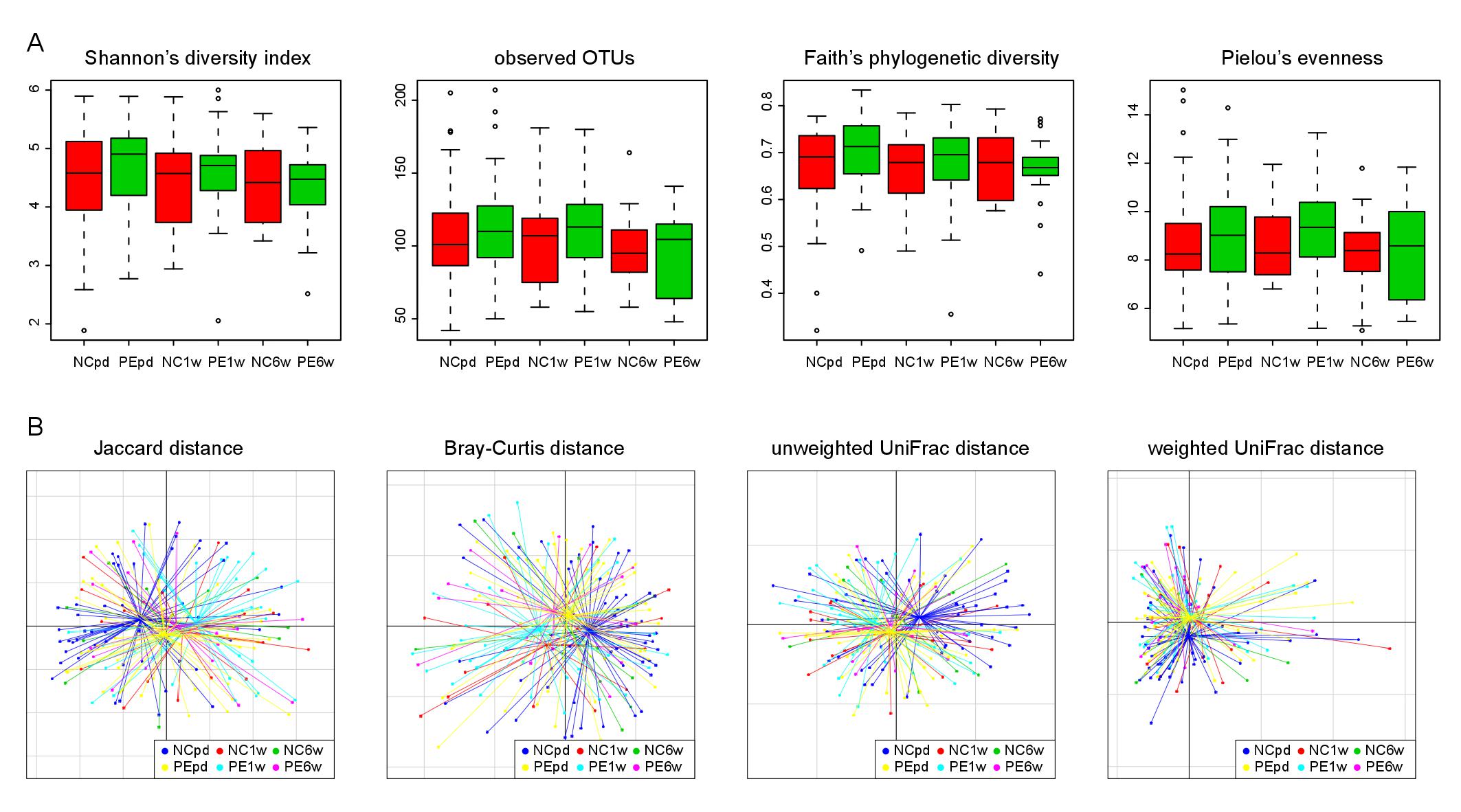

Supplement: Figure S1 — Comparison of alpha and beta diversity between PE patients and healthy controls. (A) Difference of four alpha diversity indexes between patients and controls. The boxes represent the interquartile range (IQR) between the first and third quartiles and the line inside represents the median. The whiskers denote the lowest and highest values within 1.5 times IQR from the first and third quartiles, respectively. (B) PCoA plot based on four beta diversity indexes. The first and second principal coordinates are shown. Lines connect samples (colored points) in the same group, and circles cover samples near the center of gravity for each group. [file Image_1.JPEG]
